# Acute Suppurative Thyroiditis with Thyroid Abscess: A Case Report and Review of the Literature 

**Published:** 2014-01

**Authors:** Nosrat Ghaemi, Javad Sayedi, Sepideh Bagheri

**Affiliations:** 1 *Department of Pediatrics, School of Medicine, Mashhad University of Medical sciences, Mashhad, Iran.*

**Keywords:** Abscess, Staphylococcus aureus, Thyroiditis

## Abstract

**Introduction:**

Thyroid gland is well known to resist infections by rich blood supply and lymphatic drainage, high glandular content of iodine which can be bactericidal and separation of the gland from other structures of neck. Primary thyroid abscess resulting from acute suppurative thyroiditis (AST) is an unusual type of head and neck infection and it is a rare condition in children so that progression to abscess formation is even more uncommon.

**Case Report:**

In this article we report a 9 years old girl who presented with thyroid abscess. She had fever, painful swelling in the neck, sore throat, tachycardia ,restriction of neck movements and dysphagia for 6-7 days with a history of mild fever from 10 days, prior to that. The responsible organism was found to be staphylococcus aureus. Treatment began with Intravenous antibiotics and continued with incision and drainage. Thus the process led to an uncomplicated recovery.

**Conclusion:**

Although thyroid abscess is rare, but must be considered. Most common organism that cause is staphylococcus aureus. With early diagnosis and proper treatment, it can be prevented from complications. Since this disease can be associated with anatomic abnormalities such as pyriform sinus fistula, must be roule outed.

## Introduction

Acute suppurative thyroiditis (AST) leading to thyroid abscess is a rare clinical entity (1-3). Thyroid abscess and AST represent only 0.1 to 0.7% of surgically treated thyroid pathologies (4,5).

AST specially affects patients with thyroid gland pathology such as Hashimoto’s thyroiditis or thyroid cancer and in children is associated with local anatomic defects (5,6). In particular the condition is associated with the persistence of a canal originating from the 3^th^ or 4^th^ bronchial pouch that may lead to recurrent thyroid abscess (7,8). In most cases the infection spreads to thyroid gland via piriform sinus fistula (9). The left lobe is more frequently involved (10).

If AST is left untreated it can be life threatening, resulting in 12% or higher mortality (10).The combination of a congenital sinus and acute suppurative thyroiditis was first described in Japanese literature (11). 

## Case Report

A 9 years old girl presented with fever, painful swelling in the neck, painful throat and dysphagia for 6-7 days. She also had a history of mild fever and sore throat from 10 days, prior to that.

Examination revealed a tender, warm, diffuse, midline swelling in the thyroid region, with erythema on the overlying skin. The swelling moved with deglutition. Associated findings included tachycardia and restriction of neck movements. 

There was a positive history of Brucellosis from 2 months before which was said to be completely treated. Laboratory investigations revealed leukocyte count 14300 with 70% polymorphs; hemoglobin level 12.9 gr/dl, ESR was 48 mm/hour. Blood culture was sterile. Ultraso- nography of neck revealed enlargement of the thyroid gland with decreased echogenicity.

Associated pericervical lymphadenopathy was documented. The thyroid function tests showed moderate increase of t4 level (T4:13.5 g/dl) with normal TSH (1.4 IU/ml). Barium esophagogram did not show any kind of abnormality including pyriform sinus fistula. Needle aspiration of the fluctuated mass was unsuccessful.

The diagnosis of acute suppurative thyroiditis was made and parenteral antibiotic was started on the second day of admission. The patient underwent surgical operation. 

Surgical drainage yielded about 3^cc^ thick yellow pus and a thin tube drain was inserted which was draining pus for 72 hours. 

Culture of pus yielded staphylococcus aureus. Fever subsided 2 days after the surgical drainage. Dysphagia resolved after operation so she could swallow solids without any discomfort. 

On the 8^th^ day of admission, the condition had completely subsided and she had an uncomplicated recovery.

## Discussion

Primary thyroid abscess resulting from acute suppurative thyroiditis (AST) is an unusual type of head and neck infection (12). 

Thyroid gland is well known to resist infections. The protective mechanisms include: rich blood supply and lymphatic drainage, high glandular content of iodine which can be bactericidal, separation of the gland from other structures of neck by facial planes and generation of hydrogen peroxide inside the gland as a requirement for the synthesis of thyroid hormone (10,13,14). However in some situations such as persistence of piriform sinus fistula, thyroid gland becomes susceptible to infection and abscess formation which is more commonly seen in children and young adults between 20 to 40 years of age (15). 92 % of the affected patients are children and there is no gender preference in acquiring the disease (15). Clinical features include fever, sore throat, and tenderness, anterior midline swelling in the neck, dermal erythema, dysphagia, hoarseness and limitation of head movements (16). A preceding history of respiratory tract infection may also be present (12,17). Left lobe involvement is more prevalent, than the right and tachycardia, leukocytosis and increased ESR are common with typically normal thyroid function tests (8,13). However exceptions have also been reported: In one study 12% of patients were reported to have thyrotoxicosis and 17% were reported with hypothyroidisim (18). Destruction of the thyroid gland due to bacterial invasion can cause thyroid hormone release and this may result in symptomatic thyrotoxicosis (19). Thyroid radionucloide uptake scan may be normal or show a cold nodule in the area of abscess formation. However, radionucloide scanning cannot effectively differentiate AST and sub acute thyroiditis, for both conditions can show a low 123-I uptake at initial presentation (20).

Ultrasound adequately demonstrates intra or extra-thyroid abscesses and solid or mixed lesions of the thyroid as well as adjacent inflammatory nodes (7).

CT scan can be a useful imaging modality for identifying the location of abscess but it is not essential and is only reserved for unusual occasions (21).

A barium swallow is indicated to identify the presence of a piriform sinus fistula for it has good sensitivity in detecting the presence of these fistulas (22). FNA (fine needle aspiration) can differentiate between AST and sub acute thyroiditis and also provides a good means for identifying the bacteriologic origin of the condition and thus a more precise antibiotic selection can be made (2).

 Our patient matched most of the criteria of acute suppurative thyroiditis with thyroid abscess although her T4 level was in upper limit of the normal range. 

Vocal cord paralysis as a result of a thyroid abscess is extremely rare (23,24). Dyspnea with laryngeal origin proves to be a dangerous complication of thyroid abscess (25). In rare conditions thyroid abscess may be an unusual presentation of acute tonsillitis (25). Abscess formation after FNA has been observed in immunocompromised patients (26). Organisms identified in pediatric acute suppurative thyroiditis are part of the normal oropharyngeal flora. Indigenous flora in upper respiratory tract spreads via a communicating fistula to the perithyroid space and thyroid gland (12,27).

Hence predisposition to acute suppurative thyroiditis may be due to the presence of the embryologic remnant of third or fourth pharyngeal pouch, for instance. left pyriform sinus fistula (5,6,28). 

The most important causal organisms are staphylococcus aureus, hemolytic streptococci and pnuemococci, however other organisms including anaerobic and gram-negative bacteria are also responsible (1,2,26).

Rarely, mycobacterium tuberculosis has also been reported (29).

Treatment includes parenteral antibiotics and surgical drainage in conjunction with the removal of the fistula (28,30), otherwise recurrences may occur (31).

CT-guided percutaneous catheter drainage may be an effective and safe alternative to surgical therapy (32). Ablation of the fistula can be achieved less invasively through instillation of chemocauterizing agent which has shown satisfactory results (33,34).

If the abscess is not drained, it may dissect into the neck or extend to the chest. Rupture of the abscess into the trachea or esophagus is also possible (35).

In some patients thyroiditis can result in thyroid gland destruction and it may be severe enough to cause permanent hypothyroidism. Thus follow up thyroid function studies are recommended specially in cases of more diffuse thyroiditis (36).

## Conclusion

 Although thyroid abscess is rare, but must be considered. Most common organism that cause is staphylococcus aureus. With early diagnosis and proper treatment, it can be prevented from complications. Since this disease can be associated with anatomic abnormalities such as pyriform sinus fistula, must be roule outed.

## Key Messages

Acute suppurative thyroiditis with thyroid abscess is rare in children due to inherent resistance of thyroid gland to infection but when it occurs it may suggest the presence of pyriform sinus fistula.
